# Development of indirect enzyme-linked immunosorbent assay for diagnosis of canine leptospirosis

**DOI:** 10.14202/vetworld.2017.530-535

**Published:** 2017-05-20

**Authors:** A. Sathiyamoorthy, G. Selvaraju, K. M. Palanivel, and P. Srinivasan

**Affiliations:** Department of Veterinary Preventive Medicine, Veterinary College and Research Institute, Namakkal - 637 002, Tamil Nadu, India

**Keywords:** canine leptospirosis, indirect enzyme-linked immunosorbent assay, outer membrane protein, *Leptospira canicola*, Triton X-114 extraction

## Abstract

**Aim:**

This study was taken up to develop an indirect enzyme-linked immunosorbent assay (i-ELISA) for screening antibodies against *Leptospira* spp. in canines.

**Materials and Methods:**

An i-ELISA was developed using outer membrane protein extracted from *Leptospira interrogans* serovar *canicola* used for coating the well with concentration of 0.5 µg/µl. A total of 250 serum samples from clinically affected and apparently healthy dogs were collected along with relevant epidemiological data at Teaching Veterinary Clinical Complex, Veterinary College and Research Institute, Namakkal, and subjected to i-ELISA.

**Results:**

Out of 250 sera samples, 140 (56.00%) were found to be positive by i-ELISA. All the sera samples were subjected to microagglutination test (MAT) with panel of 12 different serovars. A total of 71 (28.40%) sera samples were positivity to MAT excluding the sera samples positive to *L. interrogans* serovars *canicola* and *icterohaemorrhagiae* in vaccinated dogs. Sensitivity and specificity of i-ELISA were higher in compared with MAT was 91.54% and 58.10%, respectively.

**Conclusion:**

An indirect ELISA developed for the detection of canine antileptospiral antibodies proved to be highly sensitive, rapid and easy to perform and overcome the drawbacks of MAT.

## Introduction

Leptospirosis has been recorded in dogs throughout the major continents of the world [1-8]. Leptospirosis is a widespread zoonotic disease and is a real public health concern around the world. The disease is caused by spirochetes of the genus *Leptospira*, which comprises more than 300 serovars classified under 25 serogroups based on agglutinating antigens and is classified into multiple genom species based on DNA studies.

The diagnosis of this disease is done mainly by dark microscopic examination and by isolation of these microorganisms. The microscopic examination is done using dark-field microscopy and aids in the early diagnosis of the disease. However, it has two major drawbacks: (i) Too low concentration of leptospires (<10^4^ cells/ml) which may not be detected and (ii) artifacts such as fibrin and extrusions from cellular elements can be easily mistaken for *Leptospira* by inexperienced workers [9]. Microscopic agglutination test can be used as the gold standard test for screening serovar specific antibodies against *Leptospira* spp. [10] with high sensitivity and specificity. However, it has the following disadvantages: (i) Facilities for culturing and maintaining live leptospires are needed; (ii) the method is technically demanding and time consuming, particularly when the panel is large; (iii) antibodies may not be detectable when the causative strain is not represented in the panel or only low titer is found; (iv) the microagglutination test (MAT) cannot be standardized because live leptospires are used as antigen [11]. Immunosorbent assay simple, safe, specific, sensitive, easily automated, and suitable for the examination of a large number of sera samples in the diagnosis of Canine leptospirosis. Indirect ELISA can detect genus specific antibody but MAT does not detect [12,13].

The purpose of this study was to develop an indirect ELISA to be used as an initial screening test for the detection of the genus-specific antibodies against *Leptospira* in canine sera. The specificity and sensitivity of the indirect ELISA relative to the MAT were estimated.

## Materials and Methods

### Ethical approval

Lr.No.CPCSEA/CH/2001/5286 the CPCSEA indicates that research projects involving use of animal tissues such as blood, urine etc., from pet animal and farm animals, clinical cases and necropsy need not seek approval of CPCSEA. The current research work of A. Sathiyamoorthy, Department of Veterinary Preventive Medicine, Veterinary College and Research Institute, Namakkal involves only field cases of canine leptospirosis. Hence, the approval of ethical approval may not be required. However samples were collected as per standard collection procedure.

### Collection of sera samples and epidemiological data

The study population was a convenience sample of 250 canine serum samples submitted to the diagnostic laboratory of the Department of Veterinary Preventive Medicine, Veterinary College and Research Institute, Namakkal, Tamil Nadu. Serum samples were collected from apparently healthy and clinically affected dogs (apparently healthy dogs n=78+clinically affected dogs n=172) to rule out the canine leptospirosis which were brought to Teaching Veterinary Clinical Complex, Veterinary College and Research Institute, Namakkal, Tamil Nadu.

About 2 ml of blood was collected in a vacutainer and transported to laboratory after clot formation. The blood samples were centrifuged at 1000 × *g* for 15 min, and the serum was separated [14]. Sera samples were stored in screw capped vials at −20°C until further use.

About 12 standard serovars of *Leptospira* via *Leptospira interrogans* serovars *autumnalis, australis, ballum, canicola, hardjo, hebdomadis, javanica, pyrogen, tarassovi, icterohaemorrhagiae, pomona*, and *grippotyphosa* are maintained at the Department of Veterinary Preventive Medicine, Veterinary College and Research Institute, Namakkal.

### Triton X-114 extraction

The outer membrane proteins (OMP) from *L. interrogans* serovar *canicola* were extracted as per the method described by Cunningham *et al*. [11]. Briefly, leptospires were washed in phosphate-saline–5 mM, MgCl and then extracted in the presence of 1% protein-grade Triton X-114–150 mM NaCl–10 mM Tris (pH 8)–1 mM EDTA at 4°C. The insoluble material was removed by centrifugation at 17,000 g for 10 min. After centrifugation, 20 mM CaCl was added to the supernatant. Phase separation was performed by heating the supernatant to 37°C followed by centrifugation at 1000 g for 10 min. The detergent and aqueous phases were then separated and precipitated with acetone.

### Characterization of OMP by sodium dodecyl sulfate polyacrylamide gel electrophoresis (SDS-PAGE)

The protein profile of OMP of *L. interrogans* serovar *canicola* was carried out using a denaturating SDS-PAGE according to the method described by Laemmli [15] in a vertical electrophoresis apparatus. Amrutha *et al*. [16] observed 22.5, 29, 36, 43, 77, 93 and 112 kDa as major bands of OMP of *L. canicola* by SDS-PAGE.

### Estimation of OMP extracted from L. interrogans serovar canicola

**Figure-1:**
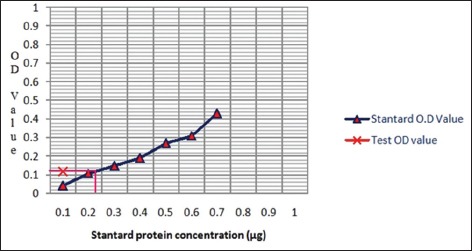
Quantification of *Leptospira interrogans* serovar *canicola* outer membrane proteins by Lowry’s method.

The protein content in the extracted samples was estimated by Lowry’s method (Figure-1) [17].

### Optimization of L. canicola OMP antigen

**Figure-2:**
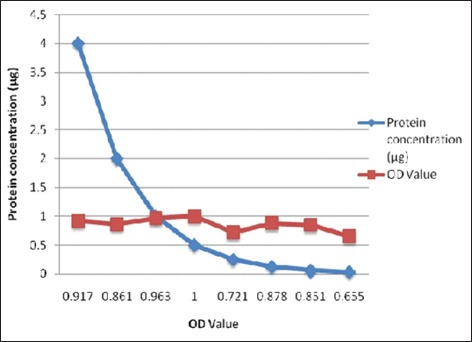
Optimization of *Leptospira interrogans* serovar *canicola* outer membrane proteins by checkerboard titration.

A checkerboard titration was carried out to determine the optimum single working dilution of OMP antigen to be used to coat the ELISA plates as per the protocol (Figure-2) [18].

### Serial dilution of L. interrogans serovar canicola OMP antigen

Serial two-fold dilutions of *L. interrogans* serovar *canicola* OMP antigen were prepared in carbonate - bicarbonate buffer (pH 9.6) to provide dilutions ranging from 4 to 0.0312 µg/well across the columns.

### Serial dilution of known positive serum sample

Known positive serum sample was serially diluted from 1:10 to 1:100 by adding phosphate buffer saline (PBS).

### Indirect ELISA

A 0.5 µg antigen in carbonate and bicarbonate buffer was coated onto each well by incubating at 4°C overnight. On the next day, the plates were washed five times with PBS with 0.05% Tween 20. 100 µl of 5% skim milk powder was added to block the uncoated sites and incubated at 37°C for 1 h. The plates were washed as above and 100 µl of 1:50 diluted test serum samples was added to individual wells in duplicates followed by incubation at 37°C for 1 h. Then, the plates were washed and 100 µl of 1:10000 diluted rabbit anti-dog HRPO conjugate was added to all the wells and again incubated at 37°C for 1 h. Then, the plates were final washed and 100 µl of freshly prepared chromogen - substrate solution containing OPD and urea H_2_O as substrate was added to each well and the plate was kept at room temperature for 10 min at dark. The enzyme-substrate reaction was stopped by adding 50 μl of 1M H_2_ SO_4_. The optical density of each of the samples was recorded at 492 nm by ELISA reader (Biorad).

### Microscopic agglutination test

The procedure suggested by Cole *et al*. [19] was carried out in round bottom 96 well microtiter plate. A volume of 50 µl of sterile PBS (pH 7.4) was added to each well. The sera samples were diluted in separate test tubes (1:25) and added to the well to make a 1:50 dilution. To these wells, constant amount of 50 µl of each antigen was added after checking the concentration of the organism under dark field mircoscopy (DFM). The final dilution after addition of antigen was ranged from 1:100 to 6400. Positive and negative controls were included in the study.

**Figure-3:**
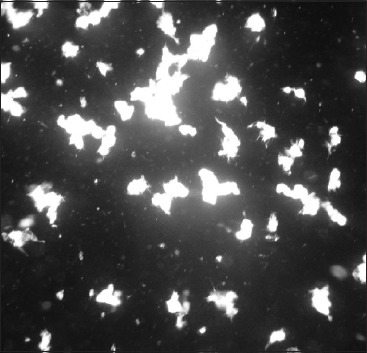
100% agglutination in microagglutination test.

The content of the microtiter plates was incubated at room temperature (37°C) for 2 h. At the end of the incubation, a loopful of sample was taken on a clean glass slide beginning from highest dilution and examined under DFM without cover glass. The end point of an agglutination reaction was taken as the highest dilution at which 50% of leptospires had agglutinated. The reciprocal of end point formed the titer. Titer of 1:100 was considered as positive titer by MAT (Figure-3) [20].

### Calculation of sensitivity and specificity

Sensitivity and specificity of the test results for each test were calculated by 2 × 2 contingency table and compared as per the method described by Smith [21].

## Result and Discussion

Confirmatory serological diagnosis of leptospirosis is usually made using the microscopic agglutination test which can detect the serovar specific antibodies but it has several disadvantages such as handling of live culture and time consuming when serovars panel is too large. Due to complexity and drawback of MAT, the search for new ELISA type of diagnostic test is essential. Hence, an indirect-ELISA was developed to screen canine anti-leptospiral antibodies in sera samples, and the results were compared with those obtained by MAT.

### Extraction of L. canicola OMP

Seven days old culture of *L. interrogans* serovar *canicola* with a concentration of 4.0×10^7^ organisms per milliliter was used to obtain OMPs that could be used as source of antigen. Kumar *et al*. [12] and Souza *et al*. [13] were used the Triton X-114 for extraction of OMP antigen from *Leptospira* serovars. *Leptospira* OMP was considered to be a potential antigen as they are conserved within the various pathogenic serovars and such proteins and their associated molecules, selectively solubilized using Triton X-114 detergent, would be of immense diagnostic value [11].

### Characterization of L. canicola OMP

The protein concentration of the extracted OMP of *L. interrogans* serovar *canicola* by SDS-PAGE and protein revealed prominent components of OMP of *L. canicola* were found to be 32, 36, 41 and 45, 112 kDa in size. Karthikeyan [14] also observed 29, 36, 43, 77, 93, and 112 kDa as major bands of OMP of *L. canicola* by SDS-PAGE.

### Quantification of L. canicola OMP

Extraction of OMP was estimated by Lowry’s method and yielded as low as concentration of 1.9 µg/µl to as high as 5.2 µg/µl. This was in accordance with the result of Kumar *et al*. [12] who extracted an average concentration of OMP was 3.66 µg/µl.

### Optimization of L. canicola OMP and determination of cutoff value for leptospirosis studies

i-ELISA was standardized by optimization of *L. canicola* OMP OD values of different antigen concentrations and serum dilution. In 1:10, 1:50 and 1:60 serum dilutions, OD value was increased from the antigen concentration of 4.0 to 1 µg/well, and thereafter OD values were declined gradually. Followed by 1:20, 1:30 and 1:40 serum dilutions, OD value was increased from the antigen concentration of 4.0 to 0.5 µg/well and thereafter OD values were declined gradually. In 1:70 serum dilution, OD value was increased from the antigen concentration of 4.0 to 2 µg/well and thereafter OD values was declined gradually and in 1:80 and above serum dilution highest OD value was observed in antigen concentration of 4.0 µg/well and followed by declined. The result revealed that antigen concentration of 0.5 µg/well at 1:30 serum dilution is suitable to screen the test serum samples. It was observed that antigen concentration of 0.5 µg/well was suitable to screen the test serum samples. Kumar *et al*. [12] also reported that antigen concentration of 0.4575 µg/well was taken as optimal antigen concentration to coat the ELISA plates for screening of test sera samples.

### Determination of cutoff OD value for leptospirosis studies

The mean OD value of control negative serum samples at 1:50 dilution was 0.276 and positive serum samples (1:50 dilution) as 1.816 using OMP concentration of 0.5 µg/well. Based on these OD values, twice the mean OD values of the negative control serum were considered as a cutoff OD value to screen the test serum samples. Therefore, any serum sample showed the OD of 0.552 and above was considered as positive for leptospirosis. Twice the mean OD values of the negative control serum were considered as a cutoff OD value to screen the test serum samples as reported by Kumar *et al*. [12]. In this study, serum sample showed the OD of 0.552 and above was considered as positive for leptospirosis.

### Seropositivity of canine leptospirosis by i-ELISA

In this study, overall seropositivity was 56.00% by developed i-ELISA. This was higher than the earlier report of Ribotta *et al*. [1], Kumar *et al*. [12], and Iwamoto *et al*. [22] who reported a seropositivity of 17.00%, 53.00%, and 3.60%, respectively.

### Seropositivity of canine leptospirosis by MAT

The MAT is the gold standard test with good specificity recommended by OIE [20] widely accepted test for diagnosis, screening of animals for international trade, and epidemiological investigations. MAT was performed in paired sera samples. Paired sera, which showed rising MAT titers that was indication of a current leptospirosis infection. Low level of titers most likely indicates vaccination reactions. In this study, overall seropositivity by MAT was 28.40% at a titer of 1:100 and above. Which was almost equal to the report of Senthil *et al*. [23] and lower than the report of Karthikeyan [14], they reported a seropositivity of 28.80% and 36.27%, respectively, in Namakkal. Several authors reported varying rate of seropositivity by MAT ranging from 11.00% [24], 71.12% [25] at different geographical areas.

### Seropositivity in healthy and clinically affected dogs

Sera samples were collected from 78 healthy dogs (Table-1). Sera were tested by MAT using a panel of 12 *Leptospira* antigens. Out of 78 sera samples, 5 (6.41%) samples were found positive for MAT. This was higher than report of Lau *et al*. [26], they reported 2.6%. Since agglutinins to MAT tend to remain in the body for a prolonged period following infection, detectable MAT titers may be present even in healthy animals [27]. Sera samples were collected from 78 healthy dogs. Sera samples were submitted to OMP of *L. interrogans* serovar *canicola* coated i-ELISA. Out of 78 sera samples, 13 (16.66%) samples were found positive in i-ELISA. Kumar *et al*. [12] also reported seropositive.

**Table-1:** Seropositive of leptospira in healthy and clinically affected dogs both MAT and i-ELISA.

S.No.	Categories	No. of animal tested	No. of positive in MAT	No. of positive in i-ELISA (%)
1.	Healthy dogs	78	5 (6.41)	66 (38.37)
2.	Clinically affected dogs	172	13 (16.66)	127 (73.83)

MAT=Microagglutination test, i-ELISA=Indirect enzyme-linked immunosorbent assay

Sera samples were collected from 172 clinically affected dogs (Table-1). Sera were tested by MAT using a panel of 12 *Leptospira* antigens. Out of 172 sera samples, 66 (38.37%) samples were found positive for MAT. This was higher than report of Lau *et al*. [26], they reported 15.8%. Sera samples were collected from 172 clinically affected dogs. Sera samples were submitted to OMP of *L. interrogans* serovar *canicola* coated i-ELISA. Out of 172 sera samples, 127 (73.83%) samples were found positive in i-ELISA. Observed seropositivity was higher than who reported 68.67% [18]. Hence, ELISA can replace MAT effectively particularly in laboratories where MAT is difficult to perform.

### Comparison of i-ELISA with MAT

In this study, sensitivity and specificity of i-ELISA compared with MAT were 91.54% and 58.10%, respectively. Observed rate of sensitivity was almost equal to the reports of Kumar *et al*. [12], Surujballi *et al*. [28], and Surujballi and Mallory [29] who reported the sensitivity of 94.94%, 95.30%, and 93.00%, respectively. Observed rate of specificity was very low when compared with the reports of Ribotta *et al*. [1], Kumar *et al*. [12], Souza *et al*. [13], Surujballi *et al*. [28], Surujballi and Mallory [29] who reported the specificity of 95.6%, 67.66%, 100%, and 94.70%, respectively. Lower specificity of i-ELISA might be due to the presence of non-agglutinating *Leptospira* antibodies that were detectable by ELISA but not by MAT, which can only detect agglutinating antibodies [30]. All the sera turned out to be MAT-negative may not actually be negative as only 12 *Leptospira* serovars were used as antigens for detection of antibody [16].

## Conclusion

In conclusion, i-ELISA developed for the detection of canine anti-leptospiral antibodies proved to be highly sensitive, rapid and easy to perform. This assay was performed with non-hazardous, highly purified and reproducible antigenic preparation which can be prepared in large quantity and could be stored for long periods. This does not require the maintenance of a constant supply of live leptospiral cultures of different serovars as in the case of MAT. This assay could be objectively interpreted and also has repeatability. Therefore, it can be recommended as a valuable screening test in routine diagnostic laboratories that do not have the facilities or expertise to perform MAT. This study revealed that age, sex, health status, and work-related activities were significantly associated with the occurrence of canine leptospirosis in this region.

## Authors’ Contributions

GS designed the research work, give the idea, share as well as supervise on practical work. AS carried out the research, collected the samples, carried out the laboratory work, and analyzed the data. KMP and PS drafted and revised the manuscript. All authors have read and approved the final manuscript.
